# Noncatalytic Functions Are Required for MPO and PON1 in Modulating the Involvement of Monocytes and Endothelial Cells in Atherosclerosis

**DOI:** 10.1155/bri/8149388

**Published:** 2026-02-15

**Authors:** Yong Li, Yunkai Liu, Yi Cheng

**Affiliations:** ^1^ Department of Epidemiology and Biostatistics, School of Public Health of Jilin University, Changchun, 130021, China; ^2^ Institute of Translational Medicine, The First Hospital of Jilin University, Changchun, China, jlu.edu.cn

**Keywords:** atherosclerosis, myeloperoxidase, noncatalytic functions, paraoxonase-1 (PON1)

## Abstract

High‐density lipoproteins (HDLs) are deeply implicated in atherosclerosis. HDL, myeloperoxidase (MPO), and paraoxonase‐1 (PON1) form a functional ternary complex where PON1 partially inhibits the MPO activity, and MPO in turn partially inactivates PON1. The activity of MPO is dependent on the concentration of hydrogen peroxide, but the extremely low concentrations of hydrogen peroxide in serums severely constrain MPO activity. PON1 has the activities of organophosphatase, arylesterase, and thiolactonase, but these hydrolase activities are extraneous to antioxidative stress. Thus, we proposed that MPO and PON1 may be involved in atherosclerosis by acting as proteins, rather than enzyme activities. Cholesterol efflux assay, ATP‐binding cassette transporter A1 (ABCA1)–dependent cholesterol efflux, and LCAT activity assay were performed. The effect of MPO, PON1, and serums from the individuals with ASCVD and healthy individuals on cholesterol efflux of human acute monocytic leukemia cell line (THP‐1 cells) was compared. Noncatalytic functions of MPO and PON1 were analyzed using recombinant proteins and neutralizing antibodies. Wound healing assay and tube formation assay were used to analyze noncatalytic functions of MPO and PON1 in modulating the involvement of human umbilical vein endothelial cells (HUVECs). We found that MPO protein decreased the cholesterol efflux; by contrast, PON1 protein increased the cholesterol efflux of THP‐1 cells. Importantly, MPO antibody partially restored cholesterol efflux, but PON1 antibody partially reduced cholesterol efflux of THP‐1 cells. Moreover, ABCA1 was necessary for controlling the involvement of MPO and PON1 in modulating cholesterol efflux of THP‐1 cells. There existed the confrontations between the noncatalytic functions of PON1 and MPO in migration of endothelial cells. Instead, MPO protein enhanced the expression of intercellular adhesion molecule‐1 (ICAM‐1) and E‐selectin of HUVECs; nonetheless, PON1 protein reduced the expression of these adhesion molecules. Of note, PON1 protein was unable to balance out the induction of MPO protein for these adhesion molecules in that the expression of these adhesion molecules generated by the combination of MPO protein and PON1 protein was similar to that of MPO. The activation of THP‐1 cells induced by MPO protein directly impaired in vitro microvascular structure via increasing the expression of IL‐6 and TNFα regulated by NF‐κB p65 of THP‐1 cells. Together, the noncatalytic functions entail MPO and PON in modulating the involvement of monocytes and endothelial cells in atherosclerosis.

## 1. Introduction

Atherosclerosis is the main cause of myocardial infarction. The early detection and early diagnosis of atherosclerosis are the prerequisites for preventing myocardial infarction. The difficulty in early diagnosis of atherosclerosis is that the histomorphology of blood vessels in the early stage of atherosclerosis is difficult to distinguish from that of healthy blood vessels. The pathological process of atherosclerosis begins with changes in lesion‐related molecules, and discovering potential disease markers from these molecules is critical for early detection and diagnosis of atherosclerosis.

High‐density lipoproteins (HDLs) are deeply implicated in atherosclerosis in that, in terms of quantity, low circulating HDLs are more associated with atherosclerotic cardiovascular disease (ASCVD); moreover, in terms of quality, the impairment of the functions of HDLs, as a major factor, leads to the early formation of atherosclerosis [1]. Thus, HDLs afford the entry points for revealing early diagnosis of atherosclerosis [2].

Of note, HDL, myeloperoxidase (MPO), and paraoxonase‐1 (PON1) form a functional ternary complex where PON1 partially inhibits the MPO activity, and MPO in turn partially inactivates PON1. This complex provides a specific regulatory platform of the function of HDLs [3]. MPO catalyzes the modification of structure and function of HDLs, worsening the effect of HDLs on promoting endothelial repair [4]. By contrast, PON1 functions as a protective factor against ASCVD via protecting HDLs from lipid oxidation [5]. Moreover, high MPO/PON1 ratio in serums acts as a potential indicator of dysfunctional HDLs and risk stratification in ASCVD [6]. In fact, there exists only a small amount of MPO present in monocytes, and MPO is lost during their maturation into macrophage [7]. Monocytes infiltrate early atherosclerotic plaques [8], indicating that these cells are facilitators for the development and rupture of atherosclerotic plaques. Interestingly, MPO induces the migration and activation of monocytes [9]; moreover, anti‐MPO antibodies attenuate the monocyte response to LPS and shape macrophage development [10]. Thus, MPO outside monocytes may function as an initiator for the involvement of monocytes in atherosclerosis. Additionally, PON1 directly suppresses the proinflammatory responses of macrophages in vitro and in vivo [11]. In terms of the HDL–MPO–PON1 complex, the functional confrontation between MPO and PON1 is capable of modulating the involvement of monocytes in atherosclerosis. Notably, the activity of MPO is dependent on the concentration of hydrogen peroxide, but the concentration is rather low in serums because hydrogen peroxide decomposes quickly [12]. PON1 has the activities of organophosphatase, arylesterase, and thiolactonase, but these hydrolase activities are extraneous to antioxidative stress [13]. However, noncatalytic functions of MPO and PON1 remain uncharted. In this study, we aimed to investigate the noncatalytic protein functions of MPO and PON1 using patient serum and cell culture models, to define their roles in cholesterol efflux, monocyte activation, and endothelial dysfunction during atherosclerosis.

## 2. Methods

### 2.1. Study Population

This study enrolled 20 individuals with ASCVD and 20 age‐ and sex‐matched healthy controls between 40 and 55 years. The individuals with ASCVD were selected from the survey of prevention and control of major chronic noncommunicable diseases in Northeast China, according to inclusion criteria ((i) with diagnosis of stenosis confirmed by coronary angiography and (ii) age between 40 and 55 years) and exclusion criteria ((i) diagnosis of active chronic inflammatory diseases [rheumatoid arthritis, inflammatory bowel disease, and systemic lupus erythematosus]; (ii) diagnosis of active malignancy; (iii) diagnosis of severe hepatic or renal dysfunction; and (iv) inability to provide informed consent). The healthy controls were selected according to criteria (without history of atherosclerosis or related cardiovascular diseases [myocardial infarction, stroke, and peripheral arterial disease] and without evidence of chronic diseases). Smoking status and the use of medications (e.g., statins and antiplatelet agents) were recorded but were not part of the exclusion criteria, allowing for the assessment of their role within a real‐world patient cohort. This study was approved by the Ethics Committee of School of Public Health of Jilin University, and written informed consent was obtained from each individual.

### 2.2. Materials

All chemicals and RPMI‐1640 medium were obtained from Dingguo Biotechnology (China). Human umbilical vein endothelial cells (HUVECs) and THP‐1 cells were sourced from FuHeng Cell Center (China). Recombinant MPO was purchased from Aibixin Biotechnology (China). Recombinant PON1 was purchased from Lianmai Biotechnology (China). Cholesterol efflux assay kit, antibodies (anti‐MPO, anti‐PON1, anti‐intercellular adhesion molecule‐1 [anti‐ICAM‐1], anti‐E‐selectin, anti‐NF‐κB p65, anti‐NF‐κB p65 [phospho S536], anti‐ApoB, and anti‐albumin), human TNF‐α ELISA kit, and human IL‐6 ELISA kit were obtained from Abcam (UK). Anti‐β‐actin was supplied by Changchun Bai’ao Trading (China). Recombinant ApoA‐I was purchased from Beyotime Biotechnology (China). Lecithin–cholesterol acyltransferase (LCAT) activity kit was from Cell Biolabs (USA). NF‐κB p65 siRNA and siRNA negative control (NC) were obtained from GenePharma (China). Chemiluminescence kit and CellTiter‐Glo Luminescent Cell Viability Assay Kit were from Promega (USA). BCA protein assay kit was from Beyotime Biotechnology (China). Matrigel was purchased from Corning (USA). Protein extraction kit was from BestBio Science (China). Lipofectamine 2000 was sourced from Invitrogen (USA). Methods using the above materials are summarized in Supporting Figure 1.

### 2.3. Isolation of ApoB‐Depleted Serums

Serums were obtained by collecting blood into tubes without anticoagulant (clot tubes). After clotting for 30–60 min at room temperature, the sample is centrifuged to separate the liquid serum from the solid clot. The clear serum is then carefully pipetted into a new, labeled tube. For storage, serums were kept refrigerated (2°C–8°C) for short‐term use (typically 1‐2 days). For long‐term preservation, it is divided into small aliquots and frozen, ideally at −80°C or below, to prevent degradation from repeated freeze‐thaw cycles. ApoB‐depleted serum was prepared on the basis of PEG 6000 [14]. Briefly, a mixture of 40 parts 20% PEG 6000 and 100 parts serum from the healthy individuals was incubated at room temperature for 20 min. ApoB‐depleted serum was then obtained by recovery of supernatant following centrifugation (10,000 rpm, 30 min, 4°C). Depletion efficiency was confirmed using western blot (Supporting Figure 2).

### 2.4. Cell Culture and Transfection

The human monocyte cell line THP‐1 was donated by peers at our laboratory. RPMI‐1640 medium was used to culture THP‐1. All cultures were supplemented with 10% (v/v) heat‐inactivated fetal calf serum, 2 mmol/L glutamine, and 1% antibiotic mixture (100 U/mL penicillin and 100 μg/mL streptomycin). HUVECs were obtained from the American Type Culture Collection (ATCC, USA), cells at passages 5–6 were used. HUVECs were cultured in RPMI‐1640 medium supplemented with 10% fetal bovine serum (FBS) and 1% penicillin–streptomycin. These cells were incubated in a CO_2_ incubator at 37°C with 5% CO_2_ in the atmosphere. THP‐1 cells were cultured in six‐well plates with 1 × 10^5^ cells/well. NF‐κB p65 siRNA and siRNA NC were transfected using Lipofectamine 2000. The concentration of rApoA‐I was 1.5 mg/mL [15]. Detailed experimental groups are listed in the legends of Figures 1, 2, and 3.

Figure 1Cholesterol efflux is involved in the confrontation between MPO and PON1 via their noncatalytic functions. (a) Serums from the healthy individuals significantly increased the cholesterol efflux of THP‐1 cells. (b) The effect of rMPO or rPON1 on the cholesterol efflux of THP‐1 cells. (c) ABCA1 was required for controlling the involvement of MPO and PON1 in modulating cholesterol efflux of THP‐1 cells. (d) LCAT was extraneous to the implication of the MPO–ApoA‐I–PON1 complex in cholesterol efflux. ^∗^ *p* < 0.05.

**Figure:**
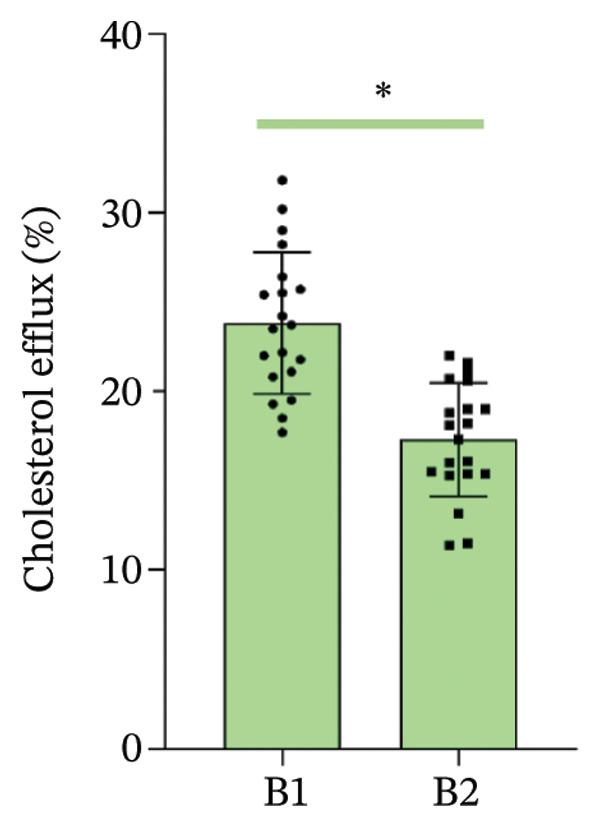
(a)

**Figure:**
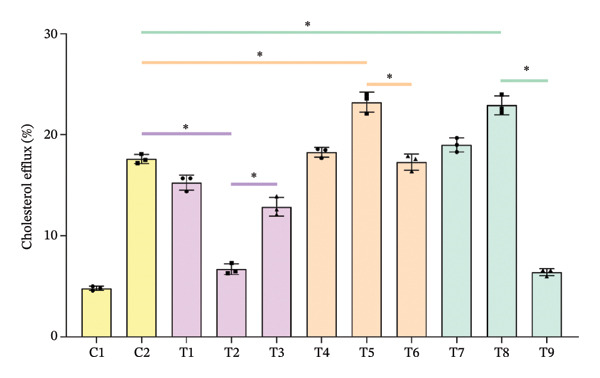
(b)

**Figure:**
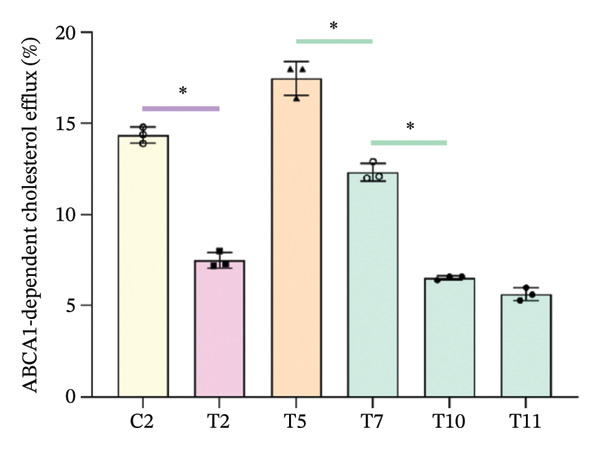
(c)

**Figure:**
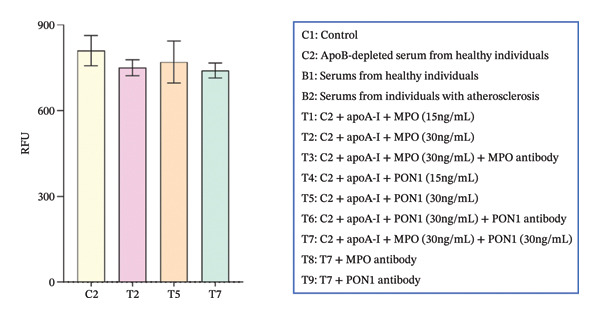
(d)

Figure 2Endothelial cell dysfunction is not directly associated with the noncatalytic functions of PON1 and MPO. (a) Noncatalytic functions of PON1 and MPO were dispensable to the viability of HUVECs. (b) rPON1 reversed the rMPO‐inhibited migration of HUVECs. (c) rMPO induced the expression of ICAM‐1 and E‐selectin of HUVECs. ^∗^ *p* < 0.05; NS: not significant.

**Figure:**
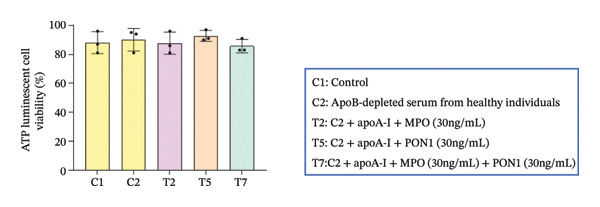
(a)

**Figure:**
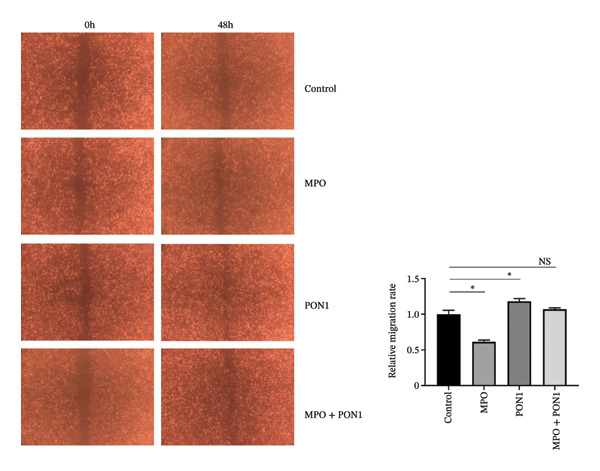
(b)

**Figure:**
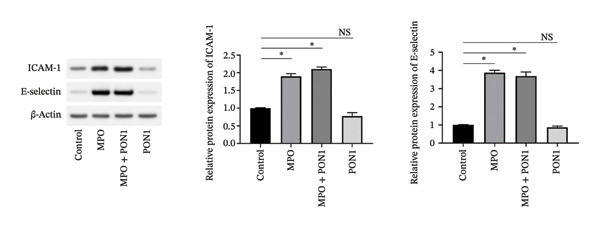
(c)

Figure 3Increasing the expression of IL‐6 and TNFα regulated by NF‐κB p65 affords the impairment of in vitro microvascular structure attributable to monocyte activation induced by MPO protein. (a) rMPO triggered the expression and secretion of IL‐6 and TNFα of THP‐1 cells. (b) Supernatants from rMPO‐treated THP‐1 cells damaged the tube formation of HUVECs. (c) NF‐κB was necessary for the expression of IL‐6 and TNFα in rMPO‐treated THP‐1 cells. ^∗^ *p* < 0.05; ^∗∗^ *p* < 0.01.

**Figure:**
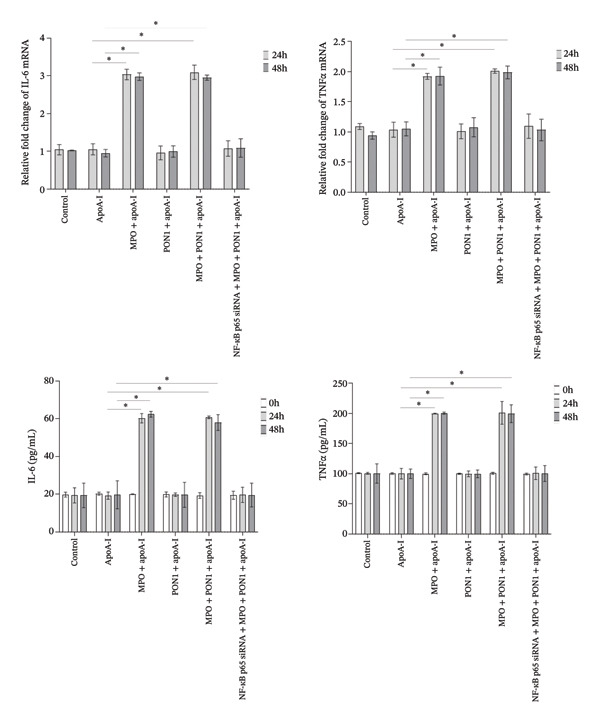
(a)

**Figure:**
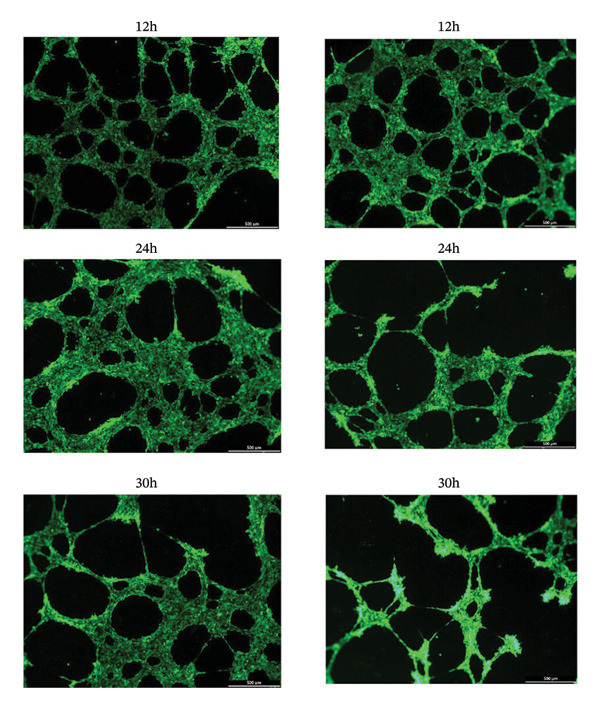
(b)

**Figure:**
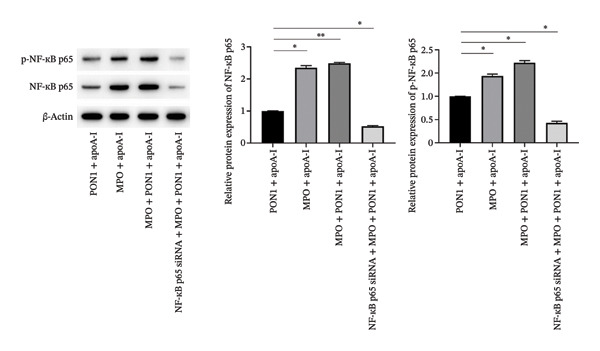
(c)

### 2.5. Cholesterol Efflux Assay

Total cholesterol (TC) efflux was performed using the cholesterol efflux assay kit. Briefly, 1 × 10^5^ THP‐1 cells/well were seeded in a 96‐well black plate with clear bottom using 100 μL media/well and incubated for 1 h to settle down. Differentiation of THP‐1 cells into adherent macrophages was induced using 100 nM phorbol 12‐myristate‐13‐acetate (PMA), and foam cell formation was induced by 130 nM IFN‐γ. These cells were incubated at 37°C with 5% CO_2_ in the atmosphere and treated using rPON1 (15 and 30 ng/mL), rMPO (15 and 30 ng/mL), and rPON1 (30 ng/mL) + rMPO (30 ng/mL), correspondingly, after labeling reagent and equilibration buffer were added. The positive control (20 μL, kit content) was added to wells of positive control, and only the serum‐free medium was added to the NC wells. After these cells were incubated for another 4 h, supernatant was transferred to a new 96‐well plate, and the cell monolayer was solubilized by 100 μL of cell lysis buffer and put on a plate shaker for 30 min at room temperature. Fluorescence of the three resulting plates was measured using Ex/Em = 482/515 nm. The percentage of cholesterol efflux was calculated by dividing the fluorescence intensity of the medium by the total of fluorescence intensity of medium and cell lysate.

### 2.6. Assay for Dependency of ATP‐Binding Cassette Transporter A1 (ABCA1)

The ABCA1‐dependent cholesterol efflux activity was similar to that in cholesterol efflux assay. The difference between ABCA1‐dependent cholesterol efflux activity and cholesterol efflux assay was that preincubation of cyclosporine A (10, 20μg/mL) was used to decrease the ABCA1 expression [16, 17].

### 2.7. LCAT Activity Assay

LCAT activity assay was performed using the commercial kit. Briefly, in a 96‐well black plate with clear bottom, samples (50 μL) were added to each well. Control wells were supplemented with 50 μL of 1X LCAT assay buffer. Iodoacetic acid, as LCAT inhibitor, was added to the appropriate samples and mixed briefly. Freshly prepared LCAT reaction reagent was used to initiate the reaction. After incubation for 8 h at 37°C, stop solution was used to stop the reaction. Fluorescence of the three resulting plates was measured using Ex/Em = 342/400 nm.

### 2.8. Wound Healing Assay

Cell migration of HUVECs was measured using wound healing assay. After transfection for 24 h, each well was scratched through the cell monolayer using a sterile pipette tip and washed with PBS. The cells were then cultured in a serum‐free medium and treated by the supernatant isolated form the culture of THP‐cells pretreated by rMPO, rPON1, or rMPO + rPON1. The scratches were scanned at the 0^th^, 18^th^, 24^th^, and 36^th^ hour. The percentage wound closure was calculated in three randomly selected fields. All assays were performed in triplicate.

### 2.9. Tube Formation Assay

Each well of a 24‐well plate was coated with 200 mL cold Matrigel, and the plate was incubated at 37°C for 1 h to gel the Matrigel. The HUVECs were cultured in the coated 24‐well plates with 10^5^ cells/well for 16 h. Then, these cells were treated by the supernatant isolated from the culture of THP‐cell pretreated by rMPO, rPON1, or rMPO + rPON1. The tube formation was observed and photographed using a fluorescence microscope (Leica, Wetzlar, Germany). The images were analyzed by Angio Tool Software (NIH, Bethesda, Maryland, USA). All assays were performed in triplicate.

### 2.10. Western Blot

Total proteins from the cultured cells were extracted using the protein extraction kit, and protein concentration was quantified using the BCA protein assay kit. Equal amount of protein samples was separated by 10% SDS‐PAGE and transferred onto nitrocellulose membranes. The membranes were blocked with 5% nonfat milk for 1 h and then incubated with anti‐ICAM‐1 antibody and anti‐E‐selectin overnight at 4°C. *β*‐Actin was used as an internal control. Afterward, washed membranes were incubated with secondary antibodies for 1 h at room temperature. The protein bands were visualized using the chemiluminescence kit and quantified using the Multi Automatic Chemiluminescence/Fluorescence Image Analysis System (China). All experiments were performed in triplicate.

### 2.11. IL‐6 and TNFα Assay

A total of 50 μL of all samples or standard was added to appropriate wells. Then, 50 μL of the IL‐6 antibody cocktail or TNF alpha antibody cocktail was added to each well. After incubating for 1 h at room temperature on a plate shaker, each well was washed and 100 μL of TMB development solution was added. Further, 100 μL of stop solution was added to each well, and absorbance at 450 nm was measured.

### 2.12. Statistical Analysis

Statistical analysis was performed using IBM SPSS 24.0 (IBM, Armonk, New York, USA). For variables with normal distribution, comparison between groups was evaluated using Student’s *t*‐test. For variables with nonnormal distribution, comparison between groups was evaluated using the rank sum test. *p* < 0.05 was considered statistically significant.

## 3. Results

### 3.1. PON1 Confronts MPO in Cholesterol Efflux via Their Noncatalytic Functions

Data obtained from all individuals included systolic blood pressure (SBP), diastolic blood pressure (DBP), TC, high‐density lipoprotein cholesterol (HDL‐C), and LDL cholesterol (LDL‐C) (Table 1). Compared with serums from the individuals with ASCVD, those from the healthy individuals significantly increased the cholesterol efflux of THP‐1 cells (*p* < 0.05) (Figure 1(a)). To further validate the involvement of noncatalytic functions of MPO and PON in affecting cholesterol efflux, we used rApoA‐I and ApoB‐depleted serum isolated from healthy individuals as a basic circumstance for rMPO and rPON1 in modulating cholesterol efflux of THP‐1 cells. We separately investigated the effect of rMPO or rPON1 on the cholesterol efflux of THP‐1 cells. We found that rMPO decreased the cholesterol efflux; by contrast, rPON1 increased the cholesterol efflux of THP‐1 cells (Figure 1(b)). Importantly, MPO antibody partially restored cholesterol efflux; moreover, PON1 antibody partially reduced cholesterol efflux of THP‐1 cells (Figure 1(b)). Thus, there existed the confrontations between PON1 and MPO in modulating the cholesterol efflux of THP‐1 cells.

**Table 1:** Baseline characteristics of healthy individuals and those with ASCVD.

	Healthy individuals (*n* = 20)	Individuals with ASCVD (*n* = 20)	*p*
Age, years	52.2 ± 4.7	55.5 ± 6.1	0.06
Male (%)	12 (60)	15 (75)	0.31
Current smoker (%)	7 (35)	14 (70)	0.02
Current alcohol drinker (%)	9 (45)	12 (60)	0.34
SBP (mm Hg)	127.6 ± 13.2	150 ± 16.1	< 0.01
DBP (mm Hg)	83.7 ± 5.6	102.6 ± 5.5	< 0.01
TC (mmol/L)	4.02 ± 0.23	5.02 ± 0.37^∗^	< 0.01
HDL‐C (mmol/L)	1.25 ± 0.33	1.02 ± 0.35^∗^	0.03
LDL‐C (mmol/L)	2.63 ± 0.27	3.59 ± 0.21^∗^	< 0.01

*Note:* Continuous data (SBP, DBP, TC, HDL‐C, and LDL‐c) are expressed as mean ± standard deviation, except for the variables ‘sex’, ‘smoking status’, and ‘alcohol drinking status’, which are presented as counts and percentages.

Abbreviations: HDL‐C, high‐density lipoprotein cholesterol; LDL‐C, low‐density lipoprotein cholesterol; TC, total cholesterol.

^∗^Asterisks represent statistical difference between healthy individuals and those with ASCVD. *p* values < 0.05 (^∗^).

The main function of ABCA1 is the efflux of intracellular free cholesterol and phospholipids across the plasma membrane to combine with Apo A‐I, forming nascent HDL‐C particles and thereby initiating the first step of reverse cholesterol transport (RCT) [18]. However, whether ABCA1 contributes to the implication of MPO and PON1 in modulating cholesterol efflux of monocytes remains uncharted. We used cyclosporine A to reduce the activity of ABCA1 because this compound directly binds to ABCA1 [16, 17]. Indeed, cyclosporine A also reduced the cholesterol efflux of THP‐1 cells treated by rMPO remarkably and significantly decreased the cholesterol efflux of THP‐1 cells treated by rPON1 or the combination of the two enzymes (*p* < 0.05) (Figure 1(b)). Thus, ABCA1 was necessary for controlling the involvement of MPO and PON1 in modulating cholesterol efflux of THP‐1 cells.

Newly secreted HDLs from the liver or intestine are discoid and require the action of LCAT in plasma to expand their core of neutral lipid and become spherical. Less is known about the implication of the MPO–ApoA‐I–PON1 complex in regulating LCAT. However, we did not find that rMPO, rPON1 protein, and the combination of rMPO protein and rPON1 protein regulated the activity of LCAT (Figure 1(c)).

### 3.2. Noncatalytic Functions of PON1 and MPO Are Irrelevant to the Dysfunction of Endothelial Cells

Dysfunction of the endothelial cells is an important contributor to the pathobiology of ASCVD [19]. Both MPO [20] and PON1 [21, 22] are versatile mediators of endothelial dysfunction. We found that rMPO, rPON1, and the combination of rMPO and rPON1 did not substantially change the viability of HUVECs (Figure 2(a)); however, rPON1 was capable of reversing the rMPO‐inhibited migration of HUVECs (Figure 2(b)). Thus, there existed the confrontations between the noncatalytic functions of PON1 and MPO in migration of endothelial cells.

Adhesion molecules are critical for the development of atherosclerosis and plaque instability. We showed that rMPO enhanced the expression of ICAM‐1 and E‐selectin of HUVECs; nonetheless, rPON1 reduced the expression of these adhesion molecules (Figure 2(c)). Of note, the expression of these adhesion molecules generated by the combination of rMPO and rPON1 was similar to that of rMPO, suggesting that PON1 cannot balance out the induction of MPO for these adhesion molecules.

### 3.3. Monocyte Activation Induced by MPO Protein Directly Impairs In Vitro Microvascular Structure via Increasing the Expression of IL‐6 and TNFα Regulated by NF‐κB p65

MPO induced the expression of ICAM‐1 and E‐selectin of HUVECs, thereby paving the way for recruitment of monocytes to endothelial cells. It is a rate‐limiting step that monocytes squeeze between endothelial cells [23]. Remarkably, this step cannot provide sufficient amounts of monocytes to form macrophages under atherosclerotic conditions because monocyte‐derived inflammatory macrophages have a short half‐life [24]. For this reason, we explored whether monocytes have extensive pathways to enter the intima. We speculated that MPO may initiate the expression and secretion of inflammatory cytokines of THP‐1 cells because MPO induces the migration and activation of monocytes [9]. Indeed, we found that rMPO triggered the expression and secretion of IL‐6 and TNFα of THP‐1 cells (Figure 3(a)); furthermore, supernatants from rMPO‐treated THP‐1 cells significantly damaged the tube formation of HUVECs (Figure 3(b)).

NF‐κB is critically involved in inflammasome activation and cytokine release [25]. rMPO significantly upregulated the levels of NF‐κB p65 and p‐NF‐κB p65 (Figure 3(c)). Knocking down NF‐κB p65 significantly downregulated the mRNA and protein levels of IL‐6 and TNFα (Figure 3(a)).

## 4. Discussion

Enzymes, compared with small molecules, have complex conformations and large sizes, thereby providing molecular scaffolds for their noncatalytic functions, including competition for protein interactions, allosteric effects on other enzymes, subcellular targeting, and DNA binding [26, 27]. The noncatalytic functions of enzymes are involved in metabolism, regulatory pathways, and pathogenesis [27]. In this study, we unveiled that the noncatalytic functions afforded MPO and PON to modulate monocytes in atherosclerosis.

The complexity of protein structures directly influences their functionality. MPO, PON1, and HDL form a functional ternary complex [3]. HDL prevents atherosclerotic plaque formation via increasing cholesterol efflux from foam cells (cholesterol‐loaded macrophages), after undergoing transcytosis of HDL from endothelial cells into the subendothelial space. Interestingly, MPO is abundant in ruptured human atherosclerotic plaques, rather than in stable atherosclerotic plaques [28]. MPO is primarily released by neutrophils, and neutrophils release hydrogen peroxide as part of their oxidative burst [29]. Thus, the major role of MPO in the ruptured human atherosclerotic plaques is the catalytic function of this enzyme. Furthermore, the major role of MPO of the functional ternary complex in serums is the noncatalytic function of this enzyme from initial to stable atherosclerotic plaques because strictly constraining MPO activity owing to the low concentration of hydrogen peroxide in serums has not been sufficiently appreciated, and erythrocytes consume hydrogen peroxide rapidly and efficiently [12]. The significant increase in MPO concentrations and decrease in PON concentrations observed in the serum of patients with acute myocardial infarction further corroborate the involvement of the noncatalytic functions of these enzymes in atherosclerosis [30]. Additionally, commercial substrates based on hydrolase activities of PON1 do not necessarily represent the physiological activity of PON1 [13]. The lack of a physiologically relevant PON1 substrate provides evidence for understanding the role of noncatalytic functions of this enzyme in atherosclerosis.

Cholesterol efflux occurs at a relatively low rate continuously, maintaining cholesterol balance within the vessel wall and preventing cholesterol deposition in healthy blood vessels, indicating that HDLs, physically, do not impair the function of endothelial cells. The alteration of MPO activity on endothelial function has been traditionally considered as downstream products and chemical modifications, including HOCl production [31], tyrosine chlorination [32], chlorinated lipid production [33], and cysteine oxidation [34]. However, these downstream products and chemical modifications result from catalytic functions of MPO. By contrast, MPO protein increases the true positive rate for ASCVD risk assessment based on the ratio of MPO protein to PON1 protein, corroborating the noncatalytic functions of these enzymes in atherosclerosis [35].

An imbalance between generation of reactive oxygen species (ROS) and antioxidant defense systems is the primary cause of endothelial dysfunction, leading to vascular damage in both metabolic and atherosclerotic diseases [36]. MPO generates highly toxic ROS under the condition of hydrogen peroxide. Obviously, the extremely low concentrations of hydrogen peroxide in serums severely constrain MPO activity. We found that MPO, PON1, and the combination of MPO and PON1 did not substantially change the viability; however, PON1 was capable of reversing the MPO‐inhibited migration of endothelial cells. Of note, excessive MPO protein was capable of increasing the expression of ICAM‐1 and E‐selectin. Our discovery corroborated that the noncatalytic functions of MPO upregulated the expression adhesion molecules of endothelial cells and provided niches attracting monocytes. Thus, the noncatalytic functions of MPO engaged in attracting monocytes via an indirect mode; by contrast, the noncatalytic functions of PON1 compromised those of MPO.

We further investigated whether the functional ternary complex formed by MPO, PON1, and HDL directly primed the activation of monocytes. Interestingly, we revealed that the noncatalytic functions of MPO were implicated in significantly upregulating the release of IL‐6 and TNFα; moreover, the activation of monocytes led to the dysfunction of endothelial cells. IL‐6 also promotes cell adhesion between monocytes and endothelial cells [37]. IL‐6 directly decreases the activity and expression of endothelial nitric oxide synthase, increasing vascular superoxide that rapidly inactivates NO, thereby limiting NO bioavailability [38]. The activation of IL‐6 trans‐signaling impairs endothelial proliferation, migration, and tube formation ability [39]; furthermore, IL‐6 promotes a sustained loss of endothelial barrier function via Janus kinase–mediated STAT3 phosphorylation and de novo protein synthesis [40]. Additionally, TNF‐α induces endothelial dysfunction [41] and endothelial cells apoptosis [42]. Unexpectedly, the noncatalytic functions of PON1 did not compromise those of MPO. Additionally, NF‐κB p65 is required for monocyte activation induced by MPO. Thus, noncatalytic functions of MPO contributed to the activation of monocytes, and activated monocytes directly caused the dysfunction and impairment of endothelial cells.

There exist limitations in this study. First, small sample size (*n* = 40) limits statistical power and generalizability. Second, heterogeneity in the ASCVD patient group (comorbidities and medications) was not accounted for in the analysis. Third, native HDL particles contain additional apolipoproteins, such as apolipoprotein A‐II and apolipoprotein A‐IV; thus, ApoB‐depleted serum plus rApoA‐I does not fully reflect the complexity of native HDL particles. Fourth, we did not include native HDL or ApoA‐I‐reconstituted HDL as controls; thus, the comparative interpretation of the relative results should be considered with caution. Fifth, measurement of eNOS activity, nitric oxide production, and other possible signaling pathways (e.g., JAK/STAT and MAPK) except NF‐κB were not investigated.

Collectively, the noncatalytic functions entail MPO and PON in modulating monocytes in atherosclerosis. Our finding provides a new dimension to this extensive line of the role of MPO and PON in atherosclerosis. The noncatalytic functions of MPO and PON1 may sustain their implications in atherosclerosis, potentially raising the constructive prospect of translational relevance neutralizing antibodies or small molecule inhibitors targeting the apoenzyme of MPO or PON.

## Funding

This study was supported by the Science Foundation of Jilin Province, 20210101464JC.

## Conflicts of Interest

The authors declare no conflicts of interest.

## Supporting Information

Additional supporting information can be found online in the supporting figures.

The workflow schematic is shown in Supporting Figure 1. The validation of the absence of ApoB is shown in Supporting Figure 2.

## Supporting information


**Supporting Information** Additional supporting information can be found online in the Supporting Information section.

## Data Availability

The data that support the findings of this study are available from the corresponding author upon reasonable request.

## References

[1] Smith J. D. , Dysfunctional HDL as a Diagnostic and Therapeutic Target, Arteriosclerosis, Thrombosis, and Vascular Biology. (2010) 30, no. 2, 151–155, 10.1161/ATVBAHA.108.179226, 2-s2.0-75149173936. 19679832 PMC2809786

[2] Rosenson R. S. , Brewer H. B. , Ansell B. J. et al., Dysfunctional HDL and Atherosclerotic Cardiovascular Disease, Nature Reviews Cardiology. (2016) 13, no. 1, 48–60, 10.1038/nrcardio.2015.124, 2-s2.0-84951574144. 26323267 PMC6245940

[3] Huang Y. , Wu Z. , Riwanto M. et al., Myeloperoxidase, Paraoxonase-1, and HDL Form a Functional Ternary Complex, Journal of Clinical Investigation. (2013) 123, no. 9, 3815–3828, 10.1172/JCI67478, 2-s2.0-84883534311. 23908111 PMC3754253

[4] Pan B. , Yu B. , Ren H. et al., High-Density Lipoprotein Nitration and Chlorination Catalyzed by Myeloperoxidase Impair Its Effect of Promoting Endothelial Repair, Free Radical Biology and Medicine. (2013) 60, 272–281, 10.1016/j.freeradbiomed.2013.02.004, 2-s2.0-84877710575. 23416364

[5] Dornas W. and Silva M. , Modulation of the Antioxidant Enzyme Paraoxonase-1 for Protection Against Cardiovascular Diseases, Nutrition, Metabolism, and Cardiovascular Diseases. (2024) 34, no. 12, 2611–2622, 10.1016/j.numecd.2024.04.005. 39277536

[6] Haraguchi Y. , Toh R. , Hasokawa M. et al., Serum myeloperoxidase/Paraoxonase 1 Ratio as Potential Indicator of Dysfunctional High-Density Lipoprotein and Risk Stratification in Coronary Artery Disease, Atherosclerosis. (2014) 234, no. 2, 288–294, 10.1016/j.atherosclerosis.2014.03.009, 2-s2.0-84900864073. 24704632

[7] Lin W. , Chen H. , Chen X. , and Guo C. , The Roles of Neutrophil-Derived Myeloperoxidase (MPO) in Diseases: The New Progress, Antioxidants. (2024) 13, no. 1, 10.3390/antiox13010132. PMC10812636 38275657

[8] Thomas G. , Tacke R. , Hedrick C. C. , and Hanna R. N. , Nonclassical Patrolling Monocyte Function in the Vasculature, Arteriosclerosis, Thrombosis, and Vascular Biology. (2015) 35, no. 6, 1306–1316, 10.1161/ATVBAHA.114.304650, 2-s2.0-84933035566. 25838429 PMC4441550

[9] Peters V. B. M. , Matheis F. , Erdmann I. et al., Myeloperoxidase Induces Monocyte Migration and Activation After Acute Myocardial Infarction, Frontiers in Immunology. (2024) 15, 10.3389/fimmu.2024.1360700. PMC11082299 38736886

[10] Popat R. J. , Hakki S. , Thakker A. et al., Anti-Myeloperoxidase Antibodies Attenuate the Monocyte Response to LPS and Shape Macrophage Development, JCI Insight. (2017) 2, no. 2, 10.1172/jci.insight.87379. PMC5256146 28138552

[11] Aharoni S. , Aviram M. , and Fuhrman B. , Paraoxonase 1 (PON1) Reduces Macrophage Inflammatory Responses, Atherosclerosis. (2013) 228, no. 2, 353–361, 10.1016/j.atherosclerosis.2013.03.005, 2-s2.0-84878113868. 23582715

[12] Orrico F. , Lopez A. C. , Saliwonczyk D. et al., The Permeability of Human Red Blood Cell Membranes to Hydrogen Peroxide is Independent of Aquaporins, Journal of Biological Chemistry. (2022) 298, no. 1, 10.1016/j.jbc.2021.101503. PMC8753180 34929164

[13] James R. W. , A Long and Winding Road: Defining the Biological Role and Clinical Importance of Paraoxonases, Clinical Chemistry and Laboratory Medicine. (2006) 44, no. 9, 1052–1059, 10.1515/CCLM.2006.207, 2-s2.0-33748546980. 16958594

[14] Li X. M. , Tang W. H. , Mosior M. K. et al., Paradoxical Association of Enhanced Cholesterol Efflux With Increased Incident Cardiovascular Risks, Arteriosclerosis, Thrombosis, and Vascular Biology. (2013) 33, no. 7, 1696–1705, 10.1161/ATVBAHA.113.301373, 2-s2.0-84879113852. 23520163 PMC3743250

[15] Chasman D. I. , Pare G. , Mora S. et al., Forty-Three Loci Associated With Plasma Lipoprotein Size, Concentration, and Cholesterol Content in Genome-Wide Analysis, PLoS Genetics. (2009) 5, no. 11, 10.1371/journal.pgen.1000730, 2-s2.0-73649103089. PMC2777390 19936222

[16] Nagao K. , Maeda M. , Manucat N. B. , and Ueda K. , Cyclosporine A and PSC833 Inhibit ABCA1 Function Via Direct Binding, Biochimica Et Biophysica Acta (BBA)-Molecular and Cell Biology of Lipids. (2013) 1831, no. 2, 398–4060, 10.1016/j.bbalip.2012.11.002, 2-s2.0-84870226769. 23153588

[17] Le Goff W. , Peng D. Q. , Settle M. , Brubaker G. , Morton R. E. , and Smith J. D. , Cyclosporin A Traps ABCA1 at the Plasma Membrane and Inhibits ABCA1-mediated Lipid Efflux to Apolipoprotein A-I, Arteriosclerosis, Thrombosis, and Vascular Biology. (2004) 24, no. 11, 2155–2161, 10.1161/01.ATV.0000144811.94581.52, 2-s2.0-8344222640. 15358601

[18] Jacobo-Albavera L. , Dominguez-Perez M. , Medina-Leyte D. J. , Gonzalez-Garrido A. , and Villarreal-Molina T. , The Role of the ATP-Binding Cassette A1 (ABCA1) in Human Disease, International Journal of Molecular Sciences. (2021) 22, no. 4, 10.3390/ijms22041593. PMC7915494 33562440

[19] Gimbrone M. A. and Garcia-Cardena G. , Endothelial Cell Dysfunction and the Pathobiology of Atherosclerosis, Circulation Research. (2016) 118, no. 4, 620–636, 10.1161/CIRCRESAHA.115.306301, 2-s2.0-84959056021. 26892962 PMC4762052

[20] Maiocchi S. L. , Ku J. , Thai T. , Chan E. , Rees M. D. , and Thomas S. R. , Myeloperoxidase: A Versatile Mediator of Endothelial Dysfunction and Therapeutic Target During Cardiovascular Disease, Pharmacology and Therapeutics. (2021) 221, 10.1016/j.pharmthera.2020.107711. 33137376

[21] Eren E. , Ellidag H. Y. , Aydin O. , and Yilmaz N. , Homocysteine, Paraoxonase-1 and Vascular Endothelial Dysfunction: Omnibus Viis Romam Pervenitur, Journal of Clinical and Diagnostic Research. (2014) 8, no. 9, CE01–CE04, 10.7860/JCDR/2014/7827.4773, 2-s2.0-84908153927. 25386429 PMC4225881

[22] Gilad D. , Atiya S. , Mozes-Autmazgin Z. et al., Paraoxonase 1 in Endothelial Cells Impairs Vasodilation Induced by Arachidonic Acid Lactone Metabolite, Biochimica et Biophysica Acta (BBA)-Molecular and Cell Biology of Lipids. (2019) 1864, no. 3, 386–393, 10.1016/j.bbalip.2018.12.008, 2-s2.0-85059652864. 30572120

[23] Muller W. A. and Weigl S. A. , Monocyte-Selective Transendothelial Migration: Dissection of the Binding and Transmigration Phases by an in Vitro Assay, Journal of Experimental Medicine. (1992) 176, no. 3, 819–828, 10.1084/jem.176.3.819, 2-s2.0-0026665291. 1512545 PMC2119361

[24] Ley K. , Miller Y. I. , and Hedrick C. C. , Monocyte and Macrophage Dynamics During Atherogenesis, Arteriosclerosis, Thrombosis, and Vascular Biology. (2011) 31, no. 7, 1506–1516, 10.1161/ATVBAHA.110.221127, 2-s2.0-79959706766. 21677293 PMC3133596

[25] Mussbacher M. , Derler M. , Basilio J. , and Schmid J. A. , NF-kappaB in Monocytes and Macrophages-An Inflammatory Master Regulator in Multitalented Immune Cells, Frontiers in Immunology. (2023) 14, 10.3389/fimmu.2023.1134661. PMC9995663 36911661

[26] Kung J. E. and Jura N. , Structural Basis for the Non-Catalytic Functions of Protein Kinases, Structure. (2016) 24, no. 1, 7–24, 10.1016/j.str.2015.10.020, 2-s2.0-84953236882. 26745528 PMC4706642

[27] Snaebjornsson M. T. and Schulze A. , Non-Canonical Functions of Enzymes Facilitate Cross-Talk Between Cell Metabolic and Regulatory Pathways, Experimental and Molecular Medicine. (2018) 50, no. 4, 1–16, 10.1038/s12276-018-0065-6, 2-s2.0-85053732576. PMC5938058 29657328

[28] Rashid I. , Maghzal G. J. , Chen Y. C. et al., Myeloperoxidase is a Potential Molecular Imaging and Therapeutic Target for the Identification and Stabilization of High-Risk Atherosclerotic Plaque, European Heart Journal. (2018) 39, no. 35, 3301–3310, 10.1093/eurheartj/ehy419, 2-s2.0-85054151171. 30219874

[29] Aratani Y. , Myeloperoxidase: Its Role for Host Defense, Inflammation, and Neutrophil Function, Archives of Biochemistry and Biophysics. (2018) 640, 47–52, 10.1016/j.abb.2018.01.004, 2-s2.0-85040680571. 29336940

[30] Ciftci H. , Gul H. F. , Sahin L. et al., Serum Myeloperoxidase, Paraoxonase, and Plasma Asprosin Concentrations in Patients With Acute Myocardial Infarction, Heliyon. (2024) 10, no. 8, 10.1016/j.heliyon.2024.e29465. PMC11043935 38665586

[31] Stocker R. , Huang A. , Jeranian E. et al., Hypochlorous Acid Impairs Endothelium-Derived Nitric Oxide Bioactivity Through a Superoxide-Dependent Mechanism, Arteriosclerosis, Thrombosis, and Vascular Biology. (2004) 24, no. 11, 2028–2033, 10.1161/01.ATV.0000143388.20994.fa, 2-s2.0-8344253531. 15331437

[32] Hazen S. L. and Heinecke J. W. , 3-Chlorotyrosine, a Specific Marker of Myeloperoxidase-Catalyzed Oxidation, is Markedly Elevated in Low Density Lipoprotein Isolated From Human Atherosclerotic Intima, Journal of Clinical Investigation. (1997) 99, no. 9, 2075–2081, 10.1172/JCI119379, 2-s2.0-0030979720. 9151778 PMC508036

[33] Meyer N. J. , Reilly J. P. , Feng R. et al., Myeloperoxidase-Derived 2-chlorofatty Acids Contribute to Human Sepsis Mortality via Acute Respiratory Distress Syndrome, JCI Insight. (2017) 2, no. 23, 10.1172/jci.insight.96432. PMC5752281 29212955

[34] Fu X. , Kassim S. Y. , Parks W. C. , and Heinecke J. W. , Hypochlorous Acid Oxygenates the Cysteine Switch Domain of Pro-Matrilysin (MMP-7). A Mechanism for Matrix Metalloproteinase Activation and Atherosclerotic Plaque Rupture by Myeloperoxidase, Journal of Biological Chemistry. (2001) 276, no. 44, 41279–41287, 10.1074/jbc.M106958200, 2-s2.0-0035798684. 11533038

[35] Variji A. , Shokri Y. , Fallahpour S. et al., The Combined Utility of Myeloperoxidase (MPO) and Paraoxonase 1 (PON1) as Two Important HDL-Associated Enzymes in Coronary Artery Disease: Which Has a Stronger Predictive Role?, Atherosclerosis. (2019) 280, 7–13, 10.1016/j.atherosclerosis.2018.11.004, 2-s2.0-85056476058. 30448568

[36] Incalza M. A. , D′Oria R. , Natalicchio A. , Perrini S. , Laviola L. , and Giorgino F. , Oxidative Stress and Reactive Oxygen Species in Endothelial Dysfunction Associated With Cardiovascular and Metabolic Diseases, Vascular Pharmacology. (2018) 100, 1–19, 10.1016/j.vph.2017.05.005, 2-s2.0-85020316170. 28579545

[37] Ohta M. , Kihara T. , Toriuchi K. et al., IL-6 Promotes Cell Adhesion in Human Endothelial Cells Via MicroRNA-126-3p Suppression, Experimental Cell Research. (2020) 393, no. 2, 10.1016/j.yexcr.2020.112094. 32439495

[38] Didion S. P. , Cellular and Oxidative Mechanisms Associated With Interleukin-6 Signaling in the Vasculature, International Journal of Molecular Sciences. (2017) 18, no. 12, 10.3390/ijms18122563, 2-s2.0-85036506620. PMC5751166 29186034

[39] Zegeye M. M. , Andersson B. , Sirsjo A. , and Ljungberg L. U. , IL-6 Trans-Signaling Impairs Sprouting Angiogenesis by Inhibiting Migration, Proliferation and Tube Formation of Human Endothelial Cells, Cells. (2020) 9, no. 6, 10.3390/cells9061414. PMC7349366 32517159

[40] Alsaffar H. , Martino N. , Garrett J. P. , and Adam A. P. , Interleukin-6 Promotes a Sustained Loss of Endothelial Barrier Function Via Janus Kinase-Mediated STAT3 Phosphorylation and De Novo Protein Synthesis, American Journal of Physiology-Cell Physiology. (2018) 314, no. 5, C589–C602, 10.1152/ajpcell.00235.2017, 2-s2.0-85050113842. 29351406

[41] Akhmedov A. , Crucet M. , Simic B. et al., Tnfalpha Induces Endothelial Dysfunction in Rheumatoid Arthritis via LOX-1 and Arginase 2: Reversal by Monoclonal TNFalpha Antibodies, Cardiovascular Research. (2022) 118, no. 1, 254–266, 10.1093/cvr/cvab005. 33483748

[42] Jiang C. , Fang X. , Jiang Y. et al., TNF-Alpha Induces Vascular Endothelial Cells Apoptosis Through Overexpressing Pregnancy Induced Noncoding RNA in Kawasaki disease Model, International Journal of Biochemistry and Cell Biology. (2016) 72, 118–124, 10.1016/j.biocel.2016.01.011, 2-s2.0-84956783059. 26794462

