# Supplementary material for: Noncatalytic Functions Are Required for MPO and PON1 in Modulating the Involvement of Monocytes and Endothelial Cells in Atherosclerosis
Source: Biochem Res Int. 2026 Feb 15;2026:8149388. doi: 10.1155/bri/8149388 (PMC12907566; doi:10.1155/bri/8149388)
Supplement: Supplementary file 1 — Supporting Information Additional supporting information can be found online in the Supporting Information section. [file BRI-2026-8149388-s001.docx]

Supplementary Figure 1. Workflow schematic

Supplementary Figure 2. Validation of the absence of ApoB
